# Efficacy of different AV7909 dose regimens in a nonclinical model of pulmonary anthrax

**DOI:** 10.1080/21645515.2023.2290345

**Published:** 2023-12-19

**Authors:** Lisa Henning, Michael Anderson, Cheryl Triplett, Tammy Smith, Kevin Boyce, Lindsay Hendey, Alex Ridenour, Jason Eng, David Schaeufele, Ehran Wilson, Carol L. Sabourin, Lily E. Adams, Tahar Babas, Lindsay Parish, Daniel Wolfe

**Affiliations:** a Battelle Biomedical Research Center, Columbus, OH, USA; b Tunnell Government Services, Inc, Supporting BARDA, Washington, DC, USA; c Oak Ridge Institute for Science and Education (ORISE) fellow at BARDA, Washington, DC, USA; d Division of CBRN Countermeasures, Biomedical Advanced Research and Development Authority (BARDA), Washington, DC, USA

**Keywords:** Anthrax, vaccine, *Bacillus anthracis*, BARDA, post-exposure prophylaxis

## Abstract

Pulmonary anthrax caused by exposure to inhaled *Bacillus anthracis*, the most lethal form of anthrax disease, is a continued military and public health concern for the United States. The vaccine AV7909, consisting of the licensed anthrax drug substance AVA adjuvanted with CpG7909, induces high levels of toxin neutralizing antibodies in healthy adults using fewer doses than AVA. This study compares the ability of one- or two-dose regimens of AV7909 to induce a protective immune response in guinea pigs challenged with a lethal dose of aerosolized *B. anthracis* spores 6 weeks after the last vaccine dose. The results indicated that AV7909 was less effective when delivered as a single dose compared to the two-dose regimen that resulted in dose-dependent protection against death. The toxin neutralizing assay (TNA) titer and anti-PA IgG responses were proportional to the protective efficacy, with a 50% TNA neutralizing factor (NF_50_) greater than 0.1 associated with survival in animals receiving two doses of vaccine. The strong protection at relatively low TNA NF_50_ titers in this guinea pig model supports the exploration of lower doses in clinical trials to determine if these protective levels of neutralizing antibodies can be achieved in humans; however, protection with a single dose may not be feasible.

## Introduction

The spores of *Bacillus anthracis*, the causative agent of anthrax, remain a biological threat to public health. The potential consequences of an attack spurred the Biomedical Advanced Research and Development Authority (BARDA), part of the Administration for Strategic Preparedness and Response (ASPR) within the US Department of Health and Human Services (HHS), to support the development and improvement of medical countermeasures that can be used to reduce the morbidity and mortality associated with inhalational anthrax. Per CDC guidelines for anthrax post-exposure prophylaxis (PEP), the response includes rapid vaccination of an exposed population after an attack to engender a protective immune response in combination with antimicrobial therapy.^1^ Mitigation strategies relying on PEP vaccination are best accomplished using a vaccine that requires a minimum number of doses to achieve rapid onset of protection.^2,3^ BioThrax® is an FDA-approved vaccine for PEP that requires a 3-dose regimen given at zero, two, and four weeks, post exposure. BioThrax is also indicated for pre-exposure prophylaxis using a 5-dose regimen that ends in an 18-month boost to induce durable anti-protective antigen (anti-PA) antibodies. While BioThrax is approved for a protective three-dose regimen,^4^ the development of a vaccine for PEP with a more rapid onset of protection following *B. anthracis* exposure and fewer required doses would enhance our ability to respond swiftly and effectively to an anthrax attack.

AV7909 is a next-generation anthrax vaccine that contains bulk Anthrax Vaccine Adsorbed (AVA) adjuvanted by CPG7909 to elicit more durable toxin neutralizing antibodies as well as protective T cell and effector cell responses after only two doses.^5^ Recently, AV7909 was licensed by the FDA as CYFENDUS (Anthrax Vaccine Adsorbed, Adjuvanted) as a two-dose regimen for PEP.^6^ AV7909 was found to elicit a protective antibody response in guinea pigs challenged with a lethal inhalational exposure of *B. anthracis* spores when the vaccine was administered prior to challenge as a two-dose regimen, and in combination with post-exposure ciprofloxacin.^7,8^ In addition, AV7909 was recently shown to induce a protective toxin neutralizing antibody response in healthy adults aged 18–50, as well as adults 66 years and older in Phase 2 clinical trials.^9,10^ These clinical trials showed that AV7909 induced a more robust immune response than Biothrax suggesting that the CpG 7909 adjuvant helps elicit the more robust immune response.^9,10^ Because a two-dose regimen of AV7909 induces a rapid and potent immune response, we hypothesized that a single-dose PEP regimen could elicit a protective immune response.

In this study, we compared the efficacy of a single AV7909 dose to a two-dose regimen in a lethal guinea pig challenge model of inhalational anthrax. Although previous anthrax countermeasures were tested in the rabbit model, lower expression of Toll-Like Receptor 9 in the rabbit diminishes the potentiation provided by the adjuvant, meaning the rabbit has no predictive value for CpG adjuvanted vaccines.^11^ In contrast, the guinea pig model described herein has been shown to accurately predict the efficacy of CpG adjuvanted vaccines against anthrax.^12^ In this study, our results indicate that a single dose of AV7909 is less effective compared to the two-dose regimen in the guinea pig anthrax model. Our results also confirm that the immune response as measured by TNA provides a robust correlate of protection against *B. anthracis* exposure.

## Materials and methods

This study was performed at the Battelle Biomedical Research Center, and the study protocol was approved by Battelle’s Institutional Animal Care and Use Committee. Virus antibody free (VAF) Hartley guinea pigs weighing 400–500 g (g) were procured from Charles River Laboratories (St. Constant, QC) and maintained in single-housing conditions. Two hundred sixteen (216; 108 male, 108 female) guinea pigs were used in the study. Prior to placement on study, guinea pigs were in good health, free of malformations, and free of clinical signs of disease. Guinea pig age was not used as a criterion for placement on this study. Animals were randomized into groups containing 24 guinea pigs each to one of four challenge cohorts (A, B, C, or D). All study events for each animal were performed on the planned study day based on their assigned day of challenge (Table S1).

On study days 0 and 28 (two-dose regimen) or only study day 28 (single-dose regimen), guinea pigs were vaccinated intramuscularly (IM; 0.5 mL) with various dilutions of AV7909^8^ or sterile saline. Blood was collected from the vena cava for immune assessment and bacteremia as described below (Table S1).

The total number of animals in each challenge cohort was divided into one of three exposure runs per day. All animals in an exposure run were exposed to the same challenge material that was prepared and characterized as previously described (*B. anthracis* Ames).^13^ Animals were exposed via nose-only aerosol exposure system (CH Technologies Tower) on study day 70.^8^ The aerosol concentration was determined by analysis of atmospheric samples collected from one of the exposure plenum ports. The samples were collected into a glass impinger. Serial dilutions of impinger samples were plated and enumerated on Tryptic Soy Agar (TSA). Rodent respiration rates and minute volumes were calculated using Guyton’s formula with mean body weights. The total inhaled dose was calculated from the product of the aerosol concentration and the total accumulated tidal volume.

All animals were observed twice daily throughout the study for clinical signs. After challenge, clinical observations were documented to include signs of illness and mortality. Animals were euthanized if they were observed with IACUC-approved euthanasia criteria. All animals were also weighed on study days 0 (baseline), 14, 28, 42, 56, 70, and 91. Necropsy, tissue collection, and histopathology were performed to confirm death due to anthrax as previously described.^8^ Blood was cultured quantitatively from all animals [(found dead or moribund) and survivors]. Briefly, a series of 1:10 serial dilutions were plated on Tryptic Soy Agar (TSA) and incubated at 37°C for 18–24 h (triplicate; starting with neat sample). Colonies consistent with *B. anthracis* morphology were then enumerated to determine bacterial burden.

The toxin neutralization assay (TNA) is designed to measure and quantify the functional ability of serum to neutralize *B. anthracis* lethal toxin activity using an *in vitro* cytotoxicity assay (J774A.1 cell line). Cell viability is determined colorimetrically using a tetrazolium salt, 3-[4, 5-dimethylthiazol-2-yl]-2, 5-diphenyltetrazolium bromide (MTT) as the reporter or signal system. The serum mediated neutralization of anthrax lethal toxin manifests as a suppression of cytotoxicity, and hence preservation of cell viability. Toxin neutralizing antibody levels, measured by effective dilution-50 (ED_50_) and neutralization factor-50 (NF_50_) were determined at the indicated time points (Table S1). NF_50_ was reported in this manuscript. The NF_50_ LOD/LOQ is 0.062.^8^

The anti-PA IgG ELISA is designed to quantify immunoglobulin class G (IgG) antibodies against *B. anthracis* PA using an ELISA in which purified recombinant PA (rPA) is used as the solid phase immobilized antigen and an enzyme-conjugated anti-gamma chain secondary antibody is used as the reporter or signal system. The guinea pig serum reference standard (generated at Battelle), with an anti-PA IgG concentration of 65.249 µg/mL was used. The anti-PA IgG ELISA LOD and LOQ is 0.155 µg/mL.^13–15^ Binding antibody levels were determined at the indicated time points (Table S1).

To evaluate survival, statistical analysis compared results from each vaccine group to the saline control as well as between the one and two-dose vaccine regimens at a common dilution. The proportion of surviving animals with Clopper-Pearson 95% confidence intervals was calculated for each group. Pairwise one-sided Boschloo’s tests were performed to determine if the proportion of surviving animals was significantly different between vaccinated groups and the saline control group while two-sided Boschloo’s tests were used to compare survival proportions between one- and two-dose vaccinated groups with common dilutions. Provided the model was able to converge without quasi-complete separation of the dilution factors for non-survivors and survivors, a logistic regression model was developed to fit the survival data from the AV7909 vaccinated groups with a continuous effect for the base-10 log-transformed dilution factor to establish efficacy of the candidate vaccine. This model was used to evaluate protective efficacy of AV7909. Kaplan–Meier estimates were plotted for the time-to-death data observed in each group and pairwise log-rank tests were performed to determine if time to death was significantly different among the groups.

Geometric means and 95% confidence intervals were calculated for quantitative bacteremia by group and time point (terminal or end of study). ANOVA models were developed to fit the base-10 log-transformed quantitative bacteremia data at each time point (terminal or end of study) to determine if there were significant differences among the groups.

Statistical analysis of the immune response data was performed to handle titer values less than the lower limit of quantitation (LLOQ) by replacing titers below LLOQ with half of the respective LLOQ. Geometric means and 95% confidence intervals along with percent coefficients of variation were calculated for ELISA titer, TNA ED_50_ and TNA NF_50_, values by group and study day. For each immune response measure (TNA ED_50_, TNA NF_50_, ELISA titer) analysis of variance (ANOVA) models were developed separately on the base-10 log-transformed data at each study day to determine if there were significant differences among the groups. Provided the models were able to converge without quasi-complete separation, logistic regression analysis was performed separately for each immune response measure (TNA ED_50_, TNA NF_50_, ELISA titer) and pre-challenge study day to test for a significant relationship between the base-10 log-transformed immune response levels and survival in the AV7909 vaccinated groups. These models included survival as the response variable and the base-10 log-transformed immune response level as a continuous covariate. The model also contained a fixed effect for vaccine group so differences in dose–response relationships could be assessed between the single and double vaccine groups. This analysis included estimates and 95% confidence intervals for the immune response levels at each pre-challenge study day necessary to achieve various survival rates.

## Results

The demonstration of efficacy for BioThrax relied on rabbit and nonhuman primate challenge models to establish antibody levels, as measured by anti-PA IgG ELISA and TNA, as the correlate of protection.^16–20^ Similarly, this study was designed to assess efficacy of one and two dose regimens of AV7909 in the guinea pig model of pulmonary anthrax disease. Groups of male and female Hartley guinea pigs were vaccinated with a two-dose (study day 0 prime, study day 28 boost) or single-dose (study day 28) regimen of AV7909 via the intramuscular route. Body weights increased over time during the post-vaccination period for all groups. Following vaccination, animals were challenged on study day 70 with a lethal aerosol exposure of *B. anthracis* Ames spores, consistent with previous challenge doses administered in the guinea pig animal model (target of 200 LD_50_ *B. anthracis* Ames spores, LD_50_ = 5.0E + 04 *B. anthracis* Ames spores).^8,12^ The mass median aerodynamic diameter ranged from 2.64 to 2.93 µm, which is consistent with lower respiratory tract deposition and systemic bacterial infection was confirmed in all animals that succumbed during the study (Table S2).^21^ Microscopic lesions observed in non-survivors were consistent with *B. anthracis* infection and confirmed death due to anthrax. Observations included evidence of septicemia with bacteria identified in multiple tissues (brain, lung, liver, lymph node, and spleen) and hemorrhage (lung and lymph node), lymphoid depletion (lymph node, spleen) as previously described (data not shown) ^12,8.^ These lesions were not observed in survivors.

**Figure 1.**
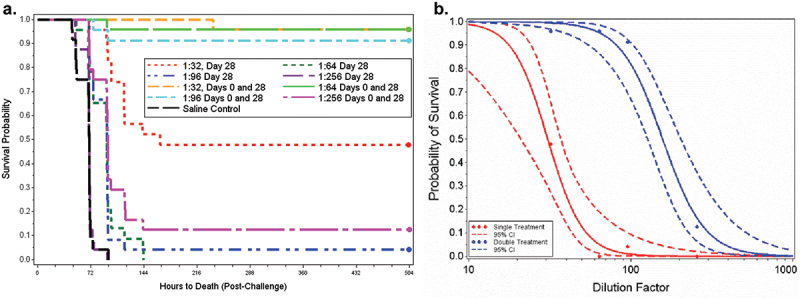
Dose-dependent survival after challenge. a. Kaplan–Meier curves representing time to death and survival data for each group vaccinated with AV7909. b. fitted logistic model for survival in the groups of vaccinated animals as a function of dilution factor and vaccine (parallel slope) overlaid on the observed data.

The primary endpoint in this study was protection against mortality after challenge as indicated by survival of animals to the end of the study period. To compare survival between treatment groups and control as well as between equivalent vaccine dilutions, the survival proportions were determined (Figure 1a) using Boschloo’s tests (one-sided to compare to control, two-sided to compare equivalent dilutions) with a Bonferroni-Holm correction for multiple comparisons. The results indicated that the proportions of surviving animals in both groups (study day 28, study day 0 and 28) vaccinated with AV7909 at the 1:32 dilution (Groups 1 and 5) and those immunized on both study days 0 and 28 at the 1:64 and 1:96 dilutions (Groups 6 and 7) were significantly greater than that in the control group (Group 9) (Table 1). Further, animals immunized on Days 0 and 28 at the 1:32, 1:64, and 1:96 dilutions (Groups 5, 6, and 7) had a significantly greater proportion of surviving animals than the respective 1:32, 1:64, and 1:96 dilution group immunized only on study day 28 (Groups 1, 2, and 3) (Table 1).

**Table 1.** Proportion of surviving animals with Clopper-Pearson 95% confidence intervals by group and results of Boschloo’s exact test comparing survival in each vaccinated group to the control group.

Group	Immunization Schedule	Vaccine	Vaccine Dilution	Number of Survivors/N	Proportion of Survivors (ClopperPearson 95% Confidence Interval)	OneSided Pairwise Boschloo’s Test *p* values^#^	Bonferroni-Holm Adjusted *p*-values^#^	TwoSided Pairwise Boschloo’s Test *p* values^	pBonferroni-Holm Adjusted *p*-values^
1	28	AV7909	1:32	11/23	0.48 (0.27, 0.69)	<.0001*	.0001*	.0002*	.0003*
2			1:64	0/23	0.00 (0.00, 0.15)	1.0000	1.0000	<.0001*	<.0001*
3			1:96	1/24	0.04 (0.00, 0.21)	.4427	1.0000	<.0001*	<.0001*
4			1:256	0/24	0.00 (0.00, 0.14)	1.0000	1.0000	.1464	.1464
5	0, 28		1:32	23/24	0.96 (0.79, 1.00)	<.0001*	<.0001*		
6			1:64	23/24	0.96 (0.79, 1.00)	<.0001*	<.0001*		
7			1:96	21/23	0.91 (0.72, 0.99)	<.0001*	<.0001*		
8			1:256	3/24	0.13 (0.03, 0.32)	.0732	.2927		
9		Control		0/24	0.00 (0.00, 0.14)				

N Number of animals.

#Comparing each vaccinated group to the control group.

^Comparing single and two-dose regimens of equivalent vaccine dilutions.

*Proportion of survivors in the vaccinated group was significantly greater than that in the control group at the 0.05 level.

To compare the efficacy of the vaccine, logistic regression analyses were performed on the survival data using a main effects model with a continuous effect for the base-10 log transformed dilution factor and a class effect for the immunization schedule was fitted (Figure 1b). The logistic curves relating survival and dilution with a common slope were significantly shifted between immunization schedules. Thus, survival increased with the two-dose regimen (study days 0 and 28) relative to the single-dose (study day 28), and the probability of survival decreased as the dilution factor increased for the vaccine.

**Figure 2.**
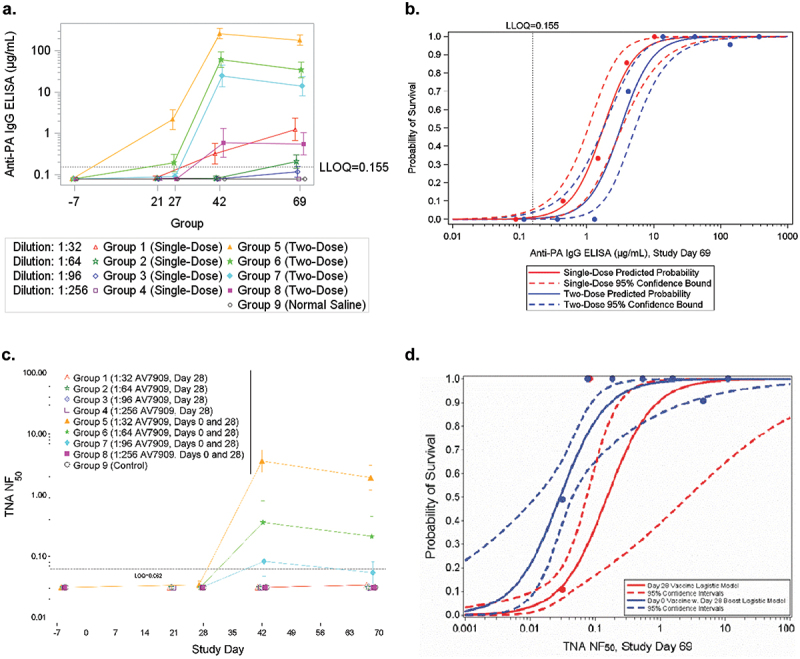
Immune correlates of protection a. geometric means and 95% confidence intervals for anti-PA IgG ELISA concentration (µg/ml). b. logistic regression model fitted to survival as a function of log-transformed anti-PA IgG ELISA concentration for vaccinated groups with a fixed effect for vaccine dose group on study day 69. c. group geometric means and 95% confidence intervals TNA NF_50_. d. fitted logistic regression model for survival in the vaccinated groups as a function of log10 TNA NF_50_ on study day 69.

As identified in animal models and supported by human clinical trials for BioThrax immunogenicity, anti-PA IgG levels and TNA response are important correlates of vaccine-mediated protection. In this study, we evaluated the immune response to AV7909 by measuring anti-PA IgG antibodies (ELISA), and neutralizing antibodies (TNA) on study days 0, 21 (single-dose groups), 27 (two-dose groups), 42, and 69. The complete data set is located in supplemental files S1 and S2. Geometric mean anti-PA IgG titers were above the lower limit of quantitation (LLOQ) in Groups 1, 2, and 5–8 on study day 69 (Figure 2a). A large boost in the immune response after the second vaccination was observed with significantly greater anti-PA IgG titers in groups treated with two doses. The magnitude of the immune response was inversely proportional to the vaccine dilution with statistical differences in anti-PA IgG titers between groups on study days 27, 42, and 69. An ANOVA model was developed separately on the base-10 log-transformed data at each study day to determine if there were significant differences among the groups. Of the two-dose groups on study day 27, the geometric mean of the anti-PA IgG titer in the 1:32 dilution group was significantly greater than that of the 1:64, 1:96, and 1:256 dilution groups; and the geometric mean of the anti-PA IgG response in the 1:64 dilution group was significantly greater than those of the 1:96 and 1:256 groups. On study days 42 and 69, after the second vaccination, the geometric means of the anti-PA IgG response in the two-dose 1:32, 1:64, and 1:96 groups were significantly greater than those of any of the single-dose groups. For the single-dose dilution groups, quantifiable anti-PA IgG titers were observed in only the 1:32 dilution on study days 42 and 69 and on study day 69 in the 1:64 dilution group. The geometric means of the anti-PA IgG titers in the single 1:32 dilution dose and the two-dose 1:256 dilution (groups 1 and 8) were significantly greater than those of the remainder of the single-dose groups. Of the two-dose dilution series, the immune response from the 1:32 dilution group was significantly greater than those of the two-dose 1:64,1:96, and 1:256 dilution groups (groups 6, 7, and 8); the geometric mean of the anti-PA IgG titers in the 1:64 dilution group was significantly greater than those of the 1:96 and 1:256 (groups 7 and 8); and the geometric mean of the anti-PA IgG titer in the 1:96 group was significantly greater than that of the 1:256 group. Furthermore, on study day 69, the geometric mean titer in the single dose 1:64 dilution group was significantly greater than those of the 1:256 group (group 4) (Table S3).

Overall, these data indicate that the 1:32 dose (group 1) was the only single-dose group to induce measurable anti-PA IgG while every two-dose group induced strong measurable anti-PA IgG concentration. To determine if there was a direct correlation between anti-PA IgG concentration and survival, a logistic regression model was fitted between survival and study day 69 anti-PA IgG concentration. The slope parameters were significantly greater than zero, indicating that the probability of survival was proportional to the anti-PA IgG concentration in both the single- and two-dose groups (Figure 2b). The estimated anti-PA IgG concentration with 95% confidence intervals for survival probabilities indicated that (Table 2), an anti-PA IgG concentration of 6.800 µg/mL on study day 69 was predictive of an 80% survival probability in the two-dose vaccination groups. An estimate for 80% survival in the single-dose groups using day 69 data calculates a titer of 3.745 µg/mL, but this value is derived from a limited number of results above the LOD. Repeat studies are necessary to thoroughly characterize potential correlates between ELISA titers and protection in the single-dose vaccine groups.

**Table 2.** Estimated anti-PA IgG concentration (µg/mL), as measured by ELISA, with 95% confidence intervals for vaccinated groups by survival probability.

Dose	Study Day	ELISA Concentration (95% Confidence Interval) by Survival Probability						
		50%	70%	75%	80%	85%	90%	95%
Single	42	0.523 (0.316, 1.091)	0.853 (0.487, 2.136)	0.986 (0.551, 2.618)	1.164 (0.633, 3.315)	1.424 (0.747, 4.421)	1.860 (0.928, 6.494)	2.864 (1.309, 12.176)
	69	1.772 (1.054, 3.365)	2.799 (1.642, 6.116)	3.206 (1.858, 7.362)	3.745 (2.136, 9.120)	4.519 (2.515, 11.885)	5.801 (3.108, 16.982)	8.680 (4.330, 30.514)
Two	42	6.237 (3.369, 10.520)	10.174 (5.936, 17.968)	11.763 (6.942, 21.330)	13.900 (8.251, 26.092)	16.982 (10.093, 33.574)	22.208 (13.017, 47.588)	34.198 (19.165, 85.408)
	69	3.217 (1.784, 5.309)	5.082 (3.041, 8.831)	5.821 (3.520, 10.387)	6.800 (4.135, 12.589)	8.204 (4.983, 16.014)	10.532 (6.310, 22.336)	15.758 (9.047, 39.039)

The TNA NF_50_ was evaluated on study days 21 (single-dose groups), 27 (two-dose groups), 42, and 69. The NF_50_ was below the limit of detection for most samples in the single-dose groups at all time points examined (Figure 2c). However, the NF_50_ in the two-dose groups at the 1:32, 1:64, and 1:96 dilution (groups 5, 6, and 7) (Figure 2c) were quantifiable on study day 42 and higher titers were generally observed with more concentrated vaccine doses. In the two-dose groups, NF_50_ titers in the 1:32 group were significantly higher than those in the 1:64 and 1:96 dilution groups and NF_50_ titers in the 1:64 dilution group were significantly higher than those in the 1:96 dilution group. On study day 69, the NF_50_ in the two-dose 1:32 dilution group was significantly higher than those in the 1:64 dilution group, and both groups were significantly higher than the 1:96 dilution group (Table S4). To determine if a similar correlation between TNA and survival that was observed for BioThrax was also observed for AV7909, a logistic regression model was fitted using survival and day 69 TNA NF_50_ from the two dose groups (Figure 2d). The graph indicates that slope parameters were significantly greater than zero, indicating the probability of survival was directly proportional to the TNA NF_50_ titers. The estimated TNA NF_50_ with 95% confidence intervals for survival probabilities indicated that a TNA NF_50_ of 0.089 on study day 69 in the two-dose vaccine groups was associated with 80% survival (Table 3). In contrast, nearly all NF_50_ results for the single-dose group were below the LOD, which resulted in a TNA NF_50_ associated with 80% survival that was not biologically meaningful (Table 3). These results, in conjunction with the anti-PA IgG concentration results, suggest that further characterization of the immune correlates in single-dose vaccine groups would require additional studies in guinea pigs, nonhuman primates, and humans or analyses of samples to determine a correlate of protection.

**Table 3.** Estimated TNA NF_50_ with 95% confidence intervals for vaccinated groups by survival probability.

Dose	Study Day	TNA NF_50_ (95% Confidence Interval) by Survival Probability					
		50%	75%	80%	85%	90%	95%
Single	42	0.185 (0.083, 2.499)	0.514 (0.167, 26.864)	0.672 (0.199, 50.296)	0.929 (0.247, 107.644)	1.428 (0.327, 296.346)	2.862 (0.513, 1526.324)
	69	0.153 (0.074, 2.662)	0.373 (0.133, 30.168)	0.471 (0.154, 57.224)	0.625 (0.185, 124.387)	0.910 (0.234, 349.444)	1.671 (0.342, 1858.488)
Two	42	<LOQ (<LOQ, <LOQ)	0.095 (<LOQ, 0.339)	0.124 (0.070, 0.607)	0.172 (0.090, 1.257)	0.264 (0.122, 3.372)	0.528 (0.196, 16.995)
	69	<LOQ (<LOQ, <LOQ)	0.070 (<LOQ, 0.243)	0.089 (<LOQ, 0.442)	0.118 (0.068, 0.933)	0.171 (0.088, 2.562)	0.315 (0.130, 13.388)

## Discussion

The concern for dissemination of *B. anthracis* spores combined with the high mortality associated with inhalational anthrax means the consequences of an anthrax attack continue to spur the development of countermeasures for prevention and treatment. After the distribution of spores in the United States postal system in 2001, the exposed population was offered a PEP regimen of antibiotics and vaccination with BioThrax.^19^ The vaccine BioThrax was approved by the FDA for a PEP indication based on the correlation between the neutralizing antibody and protection in animal models followed by clinical trials to demonstrate that the appropriate immunological response could be elicited in humans. The development of new vaccines with improved efficacy, reduced costs, and/or alternative dosing regimens to improve operational parameters remains a goal of BARDA. These efforts include the support and evaluation of AV7909, an adjuvanted anthrax vaccine intended to replace BioThrax.

In this study, the effectiveness of alternative-dosing regimens of AV7909 was compared to determine if a reduced dosing schedule could be effective in a guinea pig model of pulmonary anthrax. As in previous studies with anthrax vaccines,^8^ groups of animals were inoculated with a series of vaccine dilutions, challenged by aerosol exposure with *B. anthracis* Ames spores, and then observed for mortality for 21 days post-challenge. Other measured parameters included changes in body weight, bacteremia, and circulating levels of antibodies as measured by the TNA and ELISA for IgG against the PA antigen. The proportions of surviving animals in the groups receiving the highest dose of AV7909 (1:32, single- or two-dose regimens) or receiving 1:64 or 1:96 dilutions (two-dose regimen) were significantly greater than the proportion of surviving animals that received saline. In addition, animals receiving the two-dose AV7909 regimen at the 1:32, 1:64, and 1:96 dilutions had a significantly greater proportion of surviving animals than the respective 1:32, 1:64, and 1:96 dilution groups receiving the single-dose regimen. The ability to survive after exposure to a lethal dose of *B. anthracis* was inversely proportional to the vaccine dilution and directly proportional to the level of immune response as measured by TNA and anti-PA IgG ELISA. The survival and antibody titer data graphed as a logistic regression model further demonstrates the relationship between the probability of survival, the TNA NF_50_, and anti-PA IgG concentration by ELISA.

The results from this study clearly demonstrate that the prime boost regimen provided by two doses of AV7909 is superior to a single dose in terms of survival and immune response. The enhanced immune response may be a result of the spaced prime-boost administration, as the total antigen in the two-dose 1:96 dilution contained less material than the single-dose 1:32 vaccine but still protected a higher percentage of animals and elicited a greater immune response by both ELISA and TNA. AV7909 may prove to be preferred over BioThrax in terms of operational flexibility, dose-sparing, and accelerated treatment regimens in a PEP scenario. These data strongly suggest that a multiple dosing schedule that relies on a prime boost vaccination regimen will be required for effective protection against inhalational anthrax, and protection with a single dose may not be feasible. Future studies investigating the spacing and magnitude of these doses are best tested using clinical trials and is an area of continued investigation.

## Supplementary Material

Supplemental File 1 CLEAN 16Nov23.docx

Supplemental File 2 CLEAN 16Nov23.docx
