# Supplementary material for: Efficacy of different AV7909 dose regimens in a nonclinical model of pulmonary anthrax
Source: Hum Vaccin Immunother. 2023 Dec 19;19(3):2290345. doi: 10.1080/21645515.2023.2290345 (PMC10760354; doi:10.1080/21645515.2023.2290345)
Supplement: Supplemental File 1 CLEAN 16Nov23.docx [file KHVI_A_2290345_SM9043.docx]

| Group | Immunization Schedule (Study Days) | AV7909 Dilution | Timepoint | | |
| --- | --- | --- | --- | --- | --- |
|  |  |  | Day 21 | Day 42 | Day 69 |
| 1 | 28 | 1:32 | 0.000 | 0.027 | 0.165 |
| 1 |  |  | 0.000 | 0.266 | 0.764 |
| 1 |  |  | 0.000 | 0.053 | 0.784 |
| 1 |  |  | 0.000 | 0.732 | 1.654 |
| 1 |  |  | 0.000 | 0.000 | 0.057 |
| 1 |  |  | 0.339 | 2.630 | 4.111 |
| 1 |  |  | 0.000 | 0.418 | 3.654 |
| 1 |  |  | 0.000 | 0.000 | 0.955 |
| 1 |  |  | 0.000 | 0.024 | 0.257 |
| 1 |  |  | 0.000 | 0.660 | 5.080 |
| 1 |  |  | 0.000 | 0.122 | NS |
| 1 |  |  | 0.000 | 0.160 | 0.324 |
| 1 |  |  | 0.000 | 0.927 | 2.981 |
| 1 |  |  | NS | 0.616 | 3.162 |
| 1 |  |  | 0.000 | 0.546 | 2.305 |
| 1 |  |  | 0.000 | 4.677 | 12.960 |
| 1 |  |  | 0.000 | 0.358 | 1.574 |
| 1 |  |  | 0.000 | 0.137 | 3.223 |
| 1 |  |  | 0.000 | 0.302 | 0.312 |
| 1 |  |  | 0.000 | 0.000 | 0.220 |
| 1 |  |  | 0.000 | 0.950 | 2.268 |
| 1 |  |  | 0.000 | 0.051 | 0.193 |
| 1 |  |  | 0.014 | 4.801 | 8.176 |
| 1 |  |  | 0.000 | 1.111 | 6.771 |

| Group | Immunization Schedule (Study Days) | AV7909 Dilution | Timepoint | | |
| --- | --- | --- | --- | --- | --- |
|  |  |  | Day 21 | Day 42 | Day 69 |
| 2 | 28 | 1:64 | 0.000 | 0.047 | 0.546 |
| 2 |  |  | 0.000 | 0.000 | 0.220 |
| 2 |  |  | 0.000 | 0.039 | 0.484 |
| 2 |  |  | 0.000 | 0.142 | 0.557 |
| 2 |  |  | 0.000 | 0.000 | 0.322 |
| 2 |  |  | 0.000 | 0.000 | NS |
| 2 |  |  | 0.000 | 0.000 | 0.132 |
| 2 |  |  | 0.000 | 0.102 | 0.205 |
| 2 |  |  | 0.000 | 0.000 | 0.651 |
| 2 |  |  | 0.000 | 0.000 | 0.153 |
| 2 |  |  | 0.055 | 0.111 | 0.147 |
| 2 |  |  | 0.000 | 0.000 | 0.060 |
| 2 |  |  | 0.000 | 0.000 | 0.330 |
| 2 |  |  | 0.000 | 0.104 | 0.426 |
| 2 |  |  | 0.000 | 0.034 | 0.352 |
| 2 |  |  | 0.000 | 0.000 | 0.000 |
| 2 |  |  | 0.000 | 0.000 | 0.036 |
| 2 |  |  | 0.178 | 0.130 | 0.347 |
| 2 |  |  | 0.000 | 0.000 | 0.317 |
| 2 |  |  | 0.081 | 0.000 | 0.079 |
| 2 |  |  | 0.000 | 0.324 | 0.725 |
| 2 |  |  | 0.000 | 0.000 | 0.000 |
| 2 |  |  | 0.000 | 0.000 | 0.078 |
| 2 |  |  | 0.000 | 0.046 | 0.389 |

| Group | Immunization Schedule (Study Days) | AV7909 Dilution | Timepoint | | |
| --- | --- | --- | --- | --- | --- |
|  |  |  | Day 21 | Day 42 | Day 69 |
| 3 | 28 | 1:96 | 0.000 | 0.000 | 0.072 |
| 3 |  |  | 0.000 | 0.046 | 0.659 |
| 3 |  |  | 0.000 | 0.109 | 0.515 |
| 3 |  |  | 0.000 | 0.000 | 0.075 |
| 3 |  |  | 0.000 | 0.000 | 0.000 |
| 3 |  |  | 0.000 | 0.000 | 0.083 |
| 3 |  |  | 0.000 | 0.000 | 0.000 |
| 3 |  |  | 0.000 | 0.000 | 0.000 |
| 3 |  |  | 0.000 | 0.000 | 0.077 |
| 3 |  |  | 0.000 | 0.000 | 0.036 |
| 3 |  |  | 0.000 | 0.000 | 0.428 |
| 3 |  |  | 0.000 | 0.000 | 0.000 |
| 3 |  |  | 0.000 | 0.000 | 0.000 |
| 3 |  |  | 0.000 | 0.090 | 0.031 |
| 3 |  |  | 0.000 | 0.000 | 0.139 |
| 3 |  |  | 0.128 | 0.000 | 0.125 |
| 3 |  |  | 0.000 | 0.064 | 0.196 |
| 3 |  |  | 0.000 | 0.000 | 0.080 |
| 3 |  |  | 0.000 | 0.000 | 0.521 |
| 3 |  |  | 0.000 | 0.000 | 0.142 |
| 3 |  |  | 0.000 | 0.000 | 0.183 |
| 3 |  |  | 0.000 | 0.000 | 0.043 |
| 3 |  |  | 0.000 | 0.000 | 0.021 |
| 3 |  |  | NS | 0.023 | 0.158 |

| Group | Immunization Schedule (Study Days) | AV7909 Dilution | Timepoint | | |
| --- | --- | --- | --- | --- | --- |
|  |  |  | Day 21 | Day 42 | Day 69 |
| 4 | 28 | 1:256 | 0.000 | 0.000 | 0.023 |
| 4 |  |  | 0.000 | 0.000 | 0.000 |
| 4 |  |  | 0.000 | 0.000 | 0.000 |
| 4 |  |  | 0.000 | 0.000 | 0.000 |
| 4 |  |  | 0.000 | 0.000 | 0.019 |
| 4 |  |  | 0.015 | 0.000 | 0.048 |
| 4 |  |  | 0.000 | 0.000 | 0.041 |
| 4 |  |  | 0.000 | 0.000 | 0.000 |
| 4 |  |  | 0.000 | 0.000 | 0.000 |
| 4 |  |  | 0.000 | 0.000 | 0.071 |
| 4 |  |  | 0.000 | 0.000 | 0.000 |
| 4 |  |  | 0.000 | 0.000 | 0.000 |
| 4 |  |  | 0.000 | 0.000 | 0.039 |
| 4 |  |  | 0.054 | 0.000 | 0.119 |
| 4 |  |  | 0.000 | 0.000 | 0.000 |
| 4 |  |  | 0.187 | 0.000 | 0.000 |
| 4 |  |  | 0.000 | 0.000 | 0.000 |
| 4 |  |  | 0.000 | 0.000 | 0.030 |
| 4 |  |  | 0.000 | 0.000 | 0.119 |
| 4 |  |  | 0.000 | 0.000 | 0.026 |
| 4 |  |  | 0.000 | 0.000 | 0.000 |
| 4 |  |  | 0.000 | 0.000 | 0.000 |
| 4 |  |  | 0.000 | 0.000 | 0.000 |
| 4 |  |  | 0.000 | 0.000 | 0.000 |

| Group | Immunization Schedule (Study Days) | AV7909 Dilution | Timepoint | | | |
| --- | --- | --- | --- | --- | --- | --- |
|  |  |  | Day -7 | Day 27 | Day 42 | Day 69 |
| 5 | 0,28 | 1:32 | 0.000 | 14.553 | 651.730 | 364.616 |
| 5 |  |  | 0.000 | 3.241 | 368.850 | 281.581 |
| 5 |  |  | 0.000 | 2.809 | 240.874 | 236.730 |
| 5 |  |  | 0.000 | 1.298 | 155.678 | 101.197 |
| 5 |  |  | 0.000 | 1.296 | 184.643 | 170.287 |
| 5 |  |  | 0.000 | 4.050 | 416.760 | 283.741 |
| 5 |  |  | 0.000 | 9.779 | 513.604 | 307.021 |
| 5 |  |  | 0.000 | 1.710 | 218.285 | 159.213 |
| 5 |  |  | 0.000 | 1.389 | 240.761 | 203.070 |
| 5 |  |  | 0.000 | 1.448 | 248.633 | 124.784 |
| 5 |  |  | 0.000 | 0.144 | 93.715 | 77.878 |
| 5 |  |  | 0.000 | 3.067 | 328.441 | 163.626 |
| 5 |  |  | 0.000 | 2.404 | 260.031 | 177.500 |
| 5 |  |  | 0.000 | 4.903 | 346.506 | 159.995 |
| 5 |  |  | 0.000 | 3.274 | 496.297 | 466.869 |
| 5 |  |  | 0.000 | 2.417 | 171.471 | 148.915 |
| 5 |  |  | 0.000 | 0.321 | 104.703 | 79.636 |
| 5 |  |  | 0.000 | 11.553 | 327.445 | 231.535 |
| 5 |  |  | 0.000 | 3.030 | 672.928 | 530.233 |
| 5 |  |  | 0.000 | 3.743 | 606.095 | 215.344 |
| 5 |  |  | 0.000 | 4.902 | 590.257 | 464.487 |
| 5 |  |  | 0.000 | 1.360 | 124.838 | 139.511 |
| 5 |  |  | 0.000 | 0.000 | 44.662 | 23.692 |
| 5 |  |  | 0.000 | 7.257 | 238.306 | 132.129 |

| Group | Immunization Schedule (Study Days) | AV7909 Dilution | Timepoint | | | |
| --- | --- | --- | --- | --- | --- | --- |
|  |  |  | Day -7 | Day 27 | Day 42 | Day 69 |
| 6 | 0,28 | 1:64 | 0.000 | 0.000 | 35.893 | 25.606 |
| 6 |  |  | 0.000 | 0.075 | 143.896 | 86.228 |
| 6 |  |  | 0.000 | 0.101 | 77.426 | 47.030 |
| 6 |  |  | 0.000 | 0.350 | 96.842 | 27.814 |
| 6 |  |  | 0.000 | 0.281 | 29.786 | 12.301 |
| 6 |  |  | 0.000 | 0.000 | 21.740 | 8.574 |
| 6 |  |  | 0.000 | 0.288 | NS | 31.992 |
| 6 |  |  | 0.000 | 0.063 | 28.525 | 10.713 |
| 6 |  |  | 0.000 | 0.000 | 8.375 | 6.414 |
| 6 |  |  | 0.000 | 0.135 | 79.766 | 29.667 |
| 6 |  |  | 0.000 | 0.068 | 43.968 | 25.248 |
| 6 |  |  | 0.000 | 0.053 | 5.738 | 3.559 |
| 6 |  |  | 0.000 | 0.272 | 98.924 | 88.634 |
| 6 |  |  | 0.000 | 3.280 | 202.536 | 165.030 |
| 6 |  |  | 0.000 | 0.387 | 165.846 | 150.618 |
| 6 |  |  | 0.000 | 0.144 | 109.836 | 40.173 |
| 6 |  |  | 0.000 | 0.305 | 138.350 | 59.334 |
| 6 |  |  | 0.000 | 3.278 | 211.184 | 102.365 |
| 6 |  |  | 0.000 | 0.000 | 17.259 | 13.400 |
| 6 |  |  | 0.000 | 0.401 | 62.977 | 44.395 |
| 6 |  |  | 0.000 | 0.250 | 167.418 | 145.188 |
| 6 |  |  | 0.000 | 0.347 | 62.644 | 37.118 |
| 6 |  |  | 0.000 | 0.398 | 108.235 | 62.500 |
| 6 |  |  | 0.000 | 0.097 | 65.392 | 41.428 |

| Group | Immunization Schedule (Study Days) | AV7909 Dilution | Timepoint | | | |
| --- | --- | --- | --- | --- | --- | --- |
|  |  |  | Day -7 | Day 27 | Day 42 | Day 69 |
| 7 | 0,28 | 1:96 | 0.000 | 0.113 | 31.424 | 18.132 |
| 7 |  |  | 0.000 | 0.087 | 108.006 | 65.148 |
| 7 |  |  | 0.000 | 0.252 | 154.960 | 96.388 |
| 7 |  |  | 0.000 | 0.140 | 38.501 | 16.448 |
| 7 |  |  | 0.000 | 0.041 | 14.303 | 8.597 |
| 7 |  |  | 0.000 | 0.000 | 21.306 | NS |
| 7 |  |  | 0.000 | 0.022 | 10.149 | 5.983 |
| 7 |  |  | 0.000 | 0.059 | 119.947 | 62.100 |
| 7 |  |  | 0.000 | 0.166 | 131.112 | 65.796 |
| 7 |  |  | 0.000 | 0.000 | 0.294 | 0.464 |
| 7 |  |  | 0.000 | 0.000 | 1.713 | 0.797 |
| 7 |  |  | 0.000 | 0.000 | 9.692 | 6.780 |
| 7 |  |  | 0.000 | 0.027 | 20.724 | 10.294 |
| 7 |  |  | 0.034 | 0.000 | 10.874 | 6.339 |
| 7 |  |  | 0.000 | 0.043 | 22.481 | 20.838 |
| 7 |  |  | 0.000 | 0.189 | 34.110 | 11.889 |
| 7 |  |  | 0.000 | 0.000 | 40.583 | 17.402 |
| 7 |  |  | 0.000 | 0.100 | 104.556 | 12.702 |
| 7 |  |  | 0.000 | 0.000 | 56.874 | 33.332 |
| 7 |  |  | 0.000 | 0.000 | 13.709 | 13.305 |
| 7 |  |  | 0.000 | 0.022 | 25.193 | 12.367 |
| 7 |  |  | 0.000 | 0.175 | 85.686 | 41.708 |
| 7 |  |  | 0.000 | 0.000 | 25.450 | 18.342 |
| 7 |  |  | 0.000 | 0.122 | 16.678 | 14.092 |

| Group | Immunization Schedule (Study Days) | AV7909 Dilution | Timepoint | | | |
| --- | --- | --- | --- | --- | --- | --- |
|  |  |  | Day -7 | Day 27 | Day 42 | Day 69 |
| 8 | 0,28 | 1:256 | 0.000 | 0.000 | 5.559 | 2.920 |
| 8 |  |  | 0.000 | 0.000 | 4.426 | 1.304 |
| 8 |  |  | 0.000 | 0.000 | NS | NS |
| 8 |  |  | 0.000 | 0.000 | 0.000 | 0.065 |
| 8 |  |  | 0.000 | 0.000 | 0.058 | 0.132 |
| 8 |  |  | 0.000 | 0.000 | 0.142 | 0.177 |
| 8 |  |  | 0.000 | 0.000 | 1.244 | 1.328 |
| 8 |  |  | 0.000 | 0.000 | NS | NS |
| 8 |  |  | 0.000 | 0.000 | 0.000 | 0.062 |
| 8 |  |  | 0.000 | 0.000 | 0.087 | 0.255 |
| 8 |  |  | 0.000 | 0.000 | 0.398 | 0.281 |
| 8 |  |  | 0.000 | 0.000 | 0.033 | 0.172 |
| 8 |  |  | 0.000 | 0.000 | 1.824 | 2.226 |
| 8 |  |  | 0.000 | 0.000 | 0.217 | 0.128 |
| 8 |  |  | 0.000 | 0.000 | NS | 3.464 |
| 8 |  |  | 0.000 | 0.021 | 4.586 | 3.080 |
| 8 |  |  | 0.000 | 0.000 | 0.159 | 0.155 |
| 8 |  |  | 0.000 | 0.000 | 0.868 | 0.528 |
| 8 |  |  | 0.000 | 0.000 | 3.612 | 2.653 |
| 8 |  |  | 0.000 | 0.000 | 3.032 | 1.266 |
| 8 |  |  | 0.000 | 0.000 | 3.192 | 1.909 |
| 8 |  |  | 0.000 | 0.000 | 5.116 | 3.398 |
| 8 |  |  | 0.000 | 0.000 | 1.800 | 0.867 |
| 8 |  |  | 0.000 | 0.000 | 0.144 | 0.186 |

| Group | Immunization Schedule (Study Days) | AV7909 Dilution | Timepoint | | | |
| --- | --- | --- | --- | --- | --- | --- |
|  |  |  | Day -7 | Day 27 | Day 42 | Day 69 |
| 9 | 0,28 | Normal Saline | 0.000 | 0.000 | 0.000 | 0.000 |
| 9 |  |  | 0.000 | 0.000 | 0.000 | 0.000 |
| 9 |  |  | 0.000 | 0.000 | 0.000 | 0.000 |
| 9 |  |  | 0.000 | 0.000 | 0.000 | 0.000 |
| 9 |  |  | 0.000 | 0.000 | 0.000 | 0.000 |
| 9 |  |  | 0.000 | 0.000 | 0.000 | 0.000 |
| 9 |  |  | 0.000 | 0.000 | 0.000 | 0.000 |
| 9 |  |  | 0.000 | 0.000 | 0.000 | 0.000 |
| 9 |  |  | 0.000 | 0.000 | 0.000 | 0.000 |
| 9 |  |  | 0.000 | 0.000 | 0.000 | 0.000 |
| 9 |  |  | 0.000 | 0.000 | 0.000 | 0.000 |
| 9 |  |  | 0.000 | 0.000 | 0.000 | 0.000 |
| 9 |  |  | 0.000 | 0.000 | 0.000 | 0.000 |
| 9 |  |  | 0.000 | 0.000 | 0.000 | 0.000 |
| 9 |  |  | 0.000 | 0.000 | 0.000 | 0.000 |
| 9 |  |  | 0.000 | 0.000 | 0.000 | 0.000 |
| 9 |  |  | 0.000 | 0.000 | 0.000 | 0.000 |
| 9 |  |  | 0.000 | 0.000 | 0.000 | 0.000 |
| 9 |  |  | 0.000 | 0.000 | 0.000 | 0.000 |
| 9 |  |  | 0.000 | 0.000 | 0.000 | 0.000 |
| 9 |  |  | 0.000 | 0.000 | 0.000 | 0.000 |
| 9 |  |  | 0.000 | 0.000 | 0.000 | 0.041 |
| 9 |  |  | 0.000 | 0.000 | 0.000 | 0.000 |
| 9 |  |  | 0.000 | 0.000 | 0.000 | 0.000 |

**Supplemental File 1. Anti-PA IgG ELISA Results (µg/mL) of Each Individual Animal at Each AnalytzedTimepoint**

NS = No Sample Available
