# Supplementary material for: Efficacy of different AV7909 dose regimens in a nonclinical model of pulmonary anthrax
Source: Hum Vaccin Immunother. 2023 Dec 19;19(3):2290345. doi: 10.1080/21645515.2023.2290345 (PMC10760354; doi:10.1080/21645515.2023.2290345)
Supplement: Supplemental File 2 CLEAN 16Nov23.docx [file KHVI_A_2290345_SM9042.docx]

| Group | Immunization Schedule (Study Days) | AV7909 Dilution | ED50 | | | NF50 | | |
| --- | --- | --- | --- | --- | --- | --- | --- | --- |
|  |  |  | Time Point | | | Time Point | | |
|  |  |  | Day 21 | Day 42 | Day 69 | Day 21 | Day 42 | Day 69 |
| 1 | 28 | 1:32 | 0 | 0 | 2 | 0.000 | 0.000 | 0.003 |
| 1 |  |  | 0 | 0 | 0 | 0.000 | 0.000 | 0.000 |
| 1 |  |  | 0 | 0 | 0 | 0.000 | 0.000 | 0.000 |
| 1 |  |  | 0 | 0 | 0 | 0.000 | 0.000 | 0.000 |
| 1 |  |  | 0 | 0 | 5 | 0.000 | 0.000 | 0.006 |
| 1 |  |  | 0 | 0 | 49 | 0.000 | 0.000 | 0.093 |
| 1 |  |  | 0 | 7 | 0 | 0.000 | 0.015 | 0.000 |
| 1 |  |  | 0 | 0 | 0 | 0.000 | 0.000 | 0.000 |
| 1 |  |  | 0 | 0 | 0 | 0.000 | 0.000 | 0.000 |
| 1 |  |  | 0 | 0 | 0 | 0.000 | 0.000 | 0.000 |
| 1 |  |  | 0 | 0 | NS | 0.000 | 0.000 | NS |
| 1 |  |  | 0 | 0 | 5 | 0.000 | 0.000 | 0.007 |
| 1 |  |  | 0 | 0 | 0 | 0.000 | 0.000 | 0.000 |
| 1 |  |  | NS | 0 | 30 | NS | 0.000 | 0.072 |
| 1 |  |  | 0 | 0 | 0 | 0.000 | 0.000 | 0.000 |
| 1 |  |  | 0 | 0 | 0 | 0.000 | 0.000 | 0.000 |
| 1 |  |  | 4 | 0 | 0 | 0.011 | 0.000 | 0.000 |
| 1 |  |  | 0 | 0 | 0 | 0.000 | 0.000 | 0.000 |
| 1 |  |  | 0 | 0 | 0 | 0.000 | 0.000 | 0.000 |
| 1 |  |  | 0 | 0 | 0 | 0.000 | 0.000 | 0.000 |
| 1 |  |  | 0 | 0 | 0 | 0.000 | 0.000 | 0.000 |
| 1 |  |  | 0 | 0 | 0 | 0.000 | 0.000 | 0.000 |
| 1 |  |  | 0 | 0 | 6 | 0.000 | 0.000 | 0.017 |
| 1 |  |  | 0 | 0 | 0 | 0.000 | 0.000 | 0.000 |

| Group | Immunization Schedule (Study Days) | AV7909 Dilution | ED50 | | | NF50 | | |
| --- | --- | --- | --- | --- | --- | --- | --- | --- |
|  |  |  | Time Point | | | Time Point | | |
|  |  |  | Day 21 | Day 42 | Day 69 | Day 21 | Day 42 | Day 69 |
| 2 | 28 | 1:64 | 0 | 0 | 4 | 0.000 | 0.000 | 0.005 |
| 2 |  |  | 0 | 0 | 6 | 0.000 | 0.000 | 0.008 |
| 2 |  |  | 0 | 0 | 6 | 0.000 | 0.000 | 0.007 |
| 2 |  |  | 0 | 0 | 4 | 0.000 | 0.000 | 0.004 |
| 2 |  |  | 0 | 0 | 3 | 0.000 | 0.000 | 0.004 |
| 2 |  |  | 0 | 0 | NS | 0.000 | 0.000 | NS |
| 2 |  |  | 0 | 0 | 0 | 0.000 | 0.000 | 0.000 |
| 2 |  |  | 0 | 0 | 0 | 0.000 | 0.000 | 0.000 |
| 2 |  |  | 0 | 0 | 0 | 0.000 | 0.000 | 0.000 |
| 2 |  |  | 0 | 0 | 0 | 0.000 | 0.000 | 0.000 |
| 2 |  |  | 0 | 0 | 0 | 0.000 | 0.000 | 0.000 |
| 2 |  |  | 0 | 0 | 5 | 0.000 | 0.000 | 0.006 |
| 2 |  |  | 0 | 0 | 0 | 0.000 | 0.000 | 0.000 |
| 2 |  |  | 0 | 0 | 0 | 0.000 | 0.000 | 0.000 |
| 2 |  |  | 0 | 0 | 0 | 0.000 | 0.000 | 0.000 |
| 2 |  |  | 0 | 0 | 0 | 0.000 | 0.000 | 0.000 |
| 2 |  |  | 0 | 0 | 0 | 0.000 | 0.000 | 0.000 |
| 2 |  |  | 0 | 0 | 0 | 0.000 | 0.000 | 0.000 |
| 2 |  |  | 0 | 0 | 0 | 0.000 | 0.000 | 0.000 |
| 2 |  |  | 0 | 0 | 0 | 0.000 | 0.000 | 0.000 |
| 2 |  |  | 0 | 0 | 0 | 0.000 | 0.000 | 0.000 |
| 2 |  |  | 0 | 0 | 0 | 0.000 | 0.000 | 0.000 |
| 2 |  |  | 0 | 0 | 0 | 0.000 | 0.000 | 0.000 |
| 2 |  |  | 0 | 0 | 0 | 0.000 | 0.000 | 0.000 |

| Group | Immunization Schedule (Study Days) | AV7909 Dilution | ED50 | | | NF50 | | |
| --- | --- | --- | --- | --- | --- | --- | --- | --- |
|  |  |  | Time Point | | | Time Point | | |
|  |  |  | Day 21 | Day 42 | Day 69 | Day 21 | Day 42 | Day 69 |
| 3 | 28 | 1:96 | 0 | 0 | 0 | 0.000 | 0.000 | 0.000 |
| 3 |  |  | 0 | 0 | 3 | 0.000 | 0.000 | 0.004 |
| 3 |  |  | 0 | 0 | 0 | 0.000 | 0.000 | 0.000 |
| 3 |  |  | 0 | 0 | 2 | 0.000 | 0.000 | 0.002 |
| 3 |  |  | 0 | 0 | 0 | 0.000 | 0.000 | 0.000 |
| 3 |  |  | 0 | 0 | 0 | 0.000 | 0.000 | 0.000 |
| 3 |  |  | 0 | 0 | 0 | 0.000 | 0.000 | 0.000 |
| 3 |  |  | 0 | 0 | 0 | 0.000 | 0.000 | 0.000 |
| 3 |  |  | 0 | 0 | 0 | 0.000 | 0.000 | 0.000 |
| 3 |  |  | 0 | 0 | 3 | 0.000 | 0.000 | 0.003 |
| 3 |  |  | 0 | 0 | 0 | 0.000 | 0.000 | 0.000 |
| 3 |  |  | 0 | 0 | 5 | 0.000 | 0.000 | 0.005 |
| 3 |  |  | 0 | 0 | 0 | 0.000 | 0.000 | 0.000 |
| 3 |  |  | 0 | 0 | 0 | 0.000 | 0.000 | 0.000 |
| 3 |  |  | 0 | 0 | 0 | 0.000 | 0.000 | 0.000 |
| 3 |  |  | 0 | 0 | 0 | 0.000 | 0.000 | 0.000 |
| 3 |  |  | 0 | 0 | 8 | 0.000 | 0.000 | 0.020 |
| 3 |  |  | 0 | 0 | 0 | 0.000 | 0.000 | 0.000 |
| 3 |  |  | 0 | 0 | 0 | 0.000 | 0.000 | 0.000 |
| 3 |  |  | 0 | 0 | 0 | 0.000 | 0.000 | 0.000 |
| 3 |  |  | 0 | 0 | 0 | 0.000 | 0.000 | 0.000 |
| 3 |  |  | 0 | 0 | 0 | 0.000 | 0.000 | 0.000 |
| 3 |  |  | 0 | 0 | 0 | 0.000 | 0.000 | 0.000 |
| 3 |  |  | NS | 0 | 0 | NS | 0.000 | 0.000 |

| Group | Immunization Schedule (Study Days) | AV7909 Dilution | ED50 | | | NF50 | | |
| --- | --- | --- | --- | --- | --- | --- | --- | --- |
|  |  |  | Time Point | | | Time Point | | |
|  |  |  | Day 21 | Day 42 | Day 69 | Day 21 | Day 42 | Day 69 |
| 4 | 28 | 1:256 | 0 | 0 | 7 | 0.000 | 0.000 | 0.008 |
| 4 |  |  | 0 | 0 | 0 | 0.000 | 0.000 | 0.000 |
| 4 |  |  | 0 | 0 | 0 | 0.000 | 0.000 | 0.000 |
| 4 |  |  | 0 | 0 | 0 | 0.000 | 0.000 | 0.000 |
| 4 |  |  | 0 | 0 | 4 | 0.000 | 0.000 | 0.004 |
| 4 |  |  | 0 | 0 | 0 | 0.000 | 0.000 | 0.000 |
| 4 |  |  | 0 | 0 | 0 | 0.000 | 0.000 | 0.000 |
| 4 |  |  | 0 | 0 | 0 | 0.000 | 0.000 | 0.000 |
| 4 |  |  | 0 | 0 | 0 | 0.000 | 0.000 | 0.000 |
| 4 |  |  | 0 | 0 | 0 | 0.000 | 0.000 | 0.000 |
| 4 |  |  | 0 | 0 | 0 | 0.000 | 0.000 | 0.000 |
| 4 |  |  | 6 | 0 | 6 | 0.015 | 0.000 | 0.007 |
| 4 |  |  | 0 | 0 | 0 | 0.000 | 0.000 | 0.000 |
| 4 |  |  | 0 | 0 | 0 | 0.000 | 0.000 | 0.000 |
| 4 |  |  | 0 | 0 | 0 | 0.000 | 0.000 | 0.000 |
| 4 |  |  | 0 | 0 | 0 | 0.000 | 0.000 | 0.000 |
| 4 |  |  | 0 | 0 | 0 | 0.000 | 0.000 | 0.000 |
| 4 |  |  | 0 | 0 | 8 | 0.000 | 0.000 | 0.020 |
| 4 |  |  | 0 | 0 | 0 | 0.000 | 0.000 | 0.000 |
| 4 |  |  | 0 | 0 | 0 | 0.000 | 0.000 | 0.000 |
| 4 |  |  | 0 | 0 | 0 | 0.000 | 0.000 | 0.000 |
| 4 |  |  | 0 | 0 | 0 | 0.000 | 0.000 | 0.000 |
| 4 |  |  | 0 | 0 | 0 | 0.000 | 0.000 | 0.000 |
| 4 |  |  | 0 | 0 | 0 | 0.000 | 0.000 | 0.000 |

| Group | Immunization Schedule (Study Days) | AV7909 Dilution | ED50 | | | | NF50 | | | |
| --- | --- | --- | --- | --- | --- | --- | --- | --- | --- | --- |
|  |  |  | Time Point | | | | Time Point | | | |
|  |  |  | Day -7 | Day 27 | Day 42 | Day 69 | Day-7 | Day 27 | Day 42 | Day 69 |
| 5 | 0,28 | 1:32 | 0 | 6 | 3878 | 2077 | 0.000 | 0.015 | 9.322 | 3.972 |
| 5 |  |  | 0 | 0 | 1049 | 746 | 0.000 | 0.000 | 2.632 | 1.113 |
| 5 |  |  | 0 | 0 | 2402 | 1996 | 0.000 | 0.000 | 5.639 | 3.917 |
| 5 |  |  | 0 | 0 | 758 | 563 | 0.000 | 0.000 | 1.909 | 1.068 |
| 5 |  |  | 0 | 0 | 894 | 478 | 0.000 | 0.000 | 2.236 | 0.926 |
| 5 |  |  | 0 | 0 | 2331 | 1326 | 0.000 | 0.000 | 5.517 | 2.576 |
| 5 |  |  | 0 | 32 | 2603 | 1887 | 0.000 | 0.092 | 6.887 | 4.513 |
| 5 |  |  | 0 | 0 | 1736 | 958 | 0.000 | 0.000 | 4.435 | 2.266 |
| 5 |  |  | 0 | 0 | 776 | 487 | 0.000 | 0.000 | 1.702 | 1.150 |
| 5 |  |  | 0 | 0 | 1655 | 536 | 0.000 | 0.000 | 4.194 | 1.279 |
| 5 |  |  | 0 | 0 | 305 | 157 | 0.000 | 0.000 | 0.629 | 0.298 |
| 5 |  |  | 0 | 0 | 2597 | 893 | 0.000 | 0.000 | 7.267 | 1.962 |
| 5 |  |  | 0 | 0 | 1517 | 717 | 0.000 | 0.000 | 3.732 | 1.725 |
| 5 |  |  | 0 | 0 | 6529 | 1706 | 0.000 | 0.000 | 10.673 | 4.326 |
| 5 |  |  | 0 | 0 | 7012 | 3079 | 0.000 | 0.000 | 11.477 | 7.786 |
| 5 |  |  | 0 | 0 | 1119 | 578 | 0.000 | 0.000 | 2.573 | 1.428 |
| 5 |  |  | 0 | 0 | 233 | 146 | 0.000 | 0.000 | 0.520 | 0.361 |
| 5 |  |  | 0 | 22 | 3323 | 1775 | 0.000 | 0.053 | 8.792 | 4.490 |
| 5 |  |  | 0 | 0 | 2630 | 1401 | 0.000 | 0.000 | 4.358 | 3.686 |
| 5 |  |  | 0 | 48 | 8796 | 4027 | 0.000 | 0.112 | 14.502 | 11.102 |
| 5 |  |  | 0 | 0 | 4317 | 1988 | 0.000 | 0.000 | 7.085 | 5.151 |
| 5 |  |  | 0 | 0 | 1137 | 803 | 0.000 | 0.000 | 3.025 | 2.203 |
| 5 |  |  | 0 | 0 | 94 | 34 | 0.000 | 0.000 | 0.262 | 0.094 |
| 5 |  |  | 0 | 0 | 1647 | 1267 | 0.000 | 0.000 | 4.348 | 3.487 |

| Group | Immunization Schedule (Study Days) | AV7909 Dilution | ED50 | | | | NF50 | | | |
| --- | --- | --- | --- | --- | --- | --- | --- | --- | --- | --- |
|  |  |  | Time Point | | | | Time Point | | | |
|  |  |  | Day -7 | Day 27 | Day 42 | Day 69 | Day-7 | Day 27 | Day 42 | Day 69 |
| 6 | 0,28 | 1:64 | 0 | 0 | 0 | 0 | 0.000 | 0.000 | 0.000 | 0.000 |
| 6 |  |  | 0 | 0 | 1136 | 737 | 0.000 | 0.000 | 2.887 | 1.446 |
| 6 |  |  | 0 | 0 | 502 | 361 | 0.000 | 0.000 | 1.279 | 0.592 |
| 6 |  |  | 0 | 0 | 195 | 51 | 0.000 | 0.000 | 0.399 | 0.096 |
| 6 |  |  | 0 | 0 | 26 | 7 | 0.000 | 0.000 | 0.068 | 0.014 |
| 6 |  |  | 0 | 0 | 47 | 14 | 0.000 | 0.000 | 0.096 | 0.026 |
| 6 |  |  | 0 | 0 | NS | 150 | 0.000 | 0.000 | NS | 0.361 |
| 6 |  |  | 0 | 0 | 20 | 0 | 0.000 | 0.000 | 0.052 | 0.000 |
| 6 |  |  | 0 | 0 | 0 | 0 | 0.000 | 0.000 | 0.000 | 0.000 |
| 6 |  |  | 0 | 0 | 107 | 39 | 0.000 | 0.000 | 0.230 | 0.072 |
| 6 |  |  | 0 | 0 | 37 | 35 | 0.000 | 0.000 | 0.091 | 0.079 |
| 6 |  |  | 0 | 0 | 0 | 2 | 0.000 | 0.000 | 0.000 | 0.002 |
| 6 |  |  | 0 | 0 | 508 | 374 | 0.000 | 0.000 | 1.339 | 0.927 |
| 6 |  |  | 0 | 0 | 3841 | 2835 | 0.000 | 0.000 | 6.271 | 7.189 |
| 6 |  |  | 0 | 0 | 336 | 180 | 0.000 | 0.000 | 0.752 | 0.441 |
| 6 |  |  | 0 | 0 | 323 | 109 | 0.000 | 0.000 | 0.745 | 0.265 |
| 6 |  |  | 0 | 0 | 2317 | 762 | 0.000 | 0.000 | 6.133 | 1.833 |
| 6 |  |  | 0 | 0 | 810 | 337 | 0.000 | 0.000 | 1.871 | 0.818 |
| 6 |  |  | 0 | 0 | 6 | 0 | 0.000 | 0.000 | 0.017 | 0.000 |
| 6 |  |  | 0 | 0 | 25 | 10 | 0.000 | 0.000 | 0.069 | 0.026 |
| 6 |  |  | 0 | 0 | 485 | 431 | 0.000 | 0.000 | 1.275 | 1.178 |
| 6 |  |  | 0 | 0 | 1954 | 1432 | 0.000 | 0.000 | 5.006 | 3.928 |
| 6 |  |  | 0 | 0 | 430 | 169 | 0.000 | 0.000 | 1.198 | 0.463 |
| 6 |  |  | 0 | 0 | 140 | 67 | 0.000 | 0.000 | 0.373 | 0.185 |

| Group | Immunization Schedule (Study Days) | AV7909 Dilution | ED50 | | | | NF50 | | | |
| --- | --- | --- | --- | --- | --- | --- | --- | --- | --- | --- |
|  |  |  | Time Point | | | | Time Point | | | |
|  |  |  | Day -7 | Day 27 | Day 42 | Day 69 | Day-7 | Day 27 | Day 42 | Day 69 |
| 7 | 0,28 | 1:96 | 0 | 0 | 0 | 14 | 0.000 | 0.000 | 0.000 | 0.020 |
| 7 |  |  | 0 | 0 | 1239 | 689 | 0.000 | 0.000 | 3.098 | 1.116 |
| 7 |  |  | 0 | 0 | 54 | 37 | 0.000 | 0.000 | 0.137 | 0.073 |
| 7 |  |  | 0 | 0 | 102 | 35 | 0.000 | 0.000 | 0.247 | 0.068 |
| 7 |  |  | 0 | 0 | 49 | 27 | 0.000 | 0.000 | 0.118 | 0.061 |
| 7 |  |  | 0 | 0 | 0 | NS | 0.000 | 0.000 | 0.000 | NS |
| 7 |  |  | 0 | 0 | 0 | 0 | 0.000 | 0.000 | 0.000 | 0.000 |
| 7 |  |  | 0 | 0 | 253 | 147 | 0.000 | 0.000 | 0.552 | 0.348 |
| 7 |  |  | 0 | 0 | 52 | 49 | 0.000 | 0.000 | 0.111 | 0.116 |
| 7 |  |  | 0 | 0 | 0 | 0 | 0.000 | 0.000 | 0.000 | 0.000 |
| 7 |  |  | 0 | 0 | 0 | 0 | 0.000 | 0.000 | 0.000 | 0.000 |
| 7 |  |  | 0 | 0 | 17 | 0 | 0.000 | 0.000 | 0.036 | 0.000 |
| 7 |  |  | 0 | 0 | 0 | 0 | 0.000 | 0.000 | 0.000 | 0.000 |
| 7 |  |  | 0 | 0 | 15 | 0 | 0.000 | 0.000 | 0.040 | 0.000 |
| 7 |  |  | 0 | 0 | 0 | 0 | 0.000 | 0.000 | 0.000 | 0.000 |
| 7 |  |  | 0 | 0 | 67 | 26 | 0.000 | 0.000 | 0.150 | 0.063 |
| 7 |  |  | 0 | 0 | 66 | 0 | 0.000 | 0.000 | 0.149 | 0.000 |
| 7 |  |  | 0 | 0 | 343 | 52 | 0.000 | 0.000 | 0.766 | 0.125 |
| 7 |  |  | 0 | 0 | 25 | 0 | 0.000 | 0.000 | 0.068 | 0.000 |
| 7 |  |  | 0 | 0 | 0 | 0 | 0.000 | 0.000 | 0.000 | 0.000 |
| 7 |  |  | 0 | 0 | 14 | 0 | 0.000 | 0.000 | 0.036 | 0.000 |
| 7 |  |  | 0 | 0 | 183 | 64 | 0.000 | 0.000 | 0.511 | 0.175 |
| 7 |  |  | 0 | 0 | 0 | 0 | 0.000 | 0.000 | 0.000 | 0.000 |
| 7 |  |  | 0 | 0 | 0 | 0 | 0.000 | 0.000 | 0.000 | 0.000 |

| Group | Immunization Schedule (Study Days) | AV7909 Dilution | ED50 | | | | NF50 | | | |
| --- | --- | --- | --- | --- | --- | --- | --- | --- | --- | --- |
|  |  |  | Time Point | | | | Time Point | | | |
|  |  |  | Day -7 | Day 27 | Day 42 | Day 69 | Day-7 | Day 27 | Day 42 | Day 69 |
| 8 | 0,28 | 1:256 | 0 | 0 | 0 | 8 | 0.000 | 0.000 | 0.000 | 0.014 |
| 8 |  |  | 0 | 0 | 0 | 0 | 0.000 | 0.000 | 0.000 | 0.000 |
| 8 |  |  | 0 | 0 | 0 | 0 | 0.000 | 0.000 | 0.000 | 0.000 |
| 8 |  |  | 0 | 0 | 0 | 0 | 0.000 | 0.000 | 0.000 | 0.000 |
| 8 |  |  | 0 | 0 | 0 | 0 | 0.000 | 0.000 | 0.000 | 0.000 |
| 8 |  |  | 0 | 0 | 0 | 5 | 0.000 | 0.000 | 0.000 | 0.006 |
| 8 |  |  | 0 | 0 | 0 | 0 | 0.000 | 0.000 | 0.000 | 0.000 |
| 8 |  |  | 0 | 0 | 0 | 0 | 0.000 | 0.000 | 0.000 | 0.000 |
| 8 |  |  | 0 | 0 | 0 | 0 | 0.000 | 0.000 | 0.000 | 0.000 |
| 8 |  |  | 0 | 0 | 0 | 5 | 0.000 | 0.000 | 0.000 | 0.005 |
| 8 |  |  | 0 | 0 | 0 | 6 | 0.000 | 0.000 | 0.000 | 0.007 |
| 8 |  |  | 0 | 0 | 0 | 4 | 0.000 | 0.000 | 0.000 | 0.005 |
| 8 |  |  | 0 | 0 | 0 | 0 | 0.000 | 0.000 | 0.000 | 0.000 |
| 8 |  |  | 0 | 0 | 0 | 0 | 0.000 | 0.000 | 0.000 | 0.000 |
| 8 |  |  | 0 | 0 | 0 | 0 | 0.000 | 0.000 | 0.000 | 0.000 |
| 8 |  |  | 0 | 0 | 0 | 0 | 0.000 | 0.000 | 0.000 | 0.000 |
| 8 |  |  | 0 | 0 | 0 | 0 | 0.000 | 0.000 | 0.000 | 0.000 |
| 8 |  |  | 0 | 0 | 0 | 0 | 0.000 | 0.000 | 0.000 | 0.000 |
| 8 |  |  | 0 | 0 | 8 | 0 | 0.000 | 0.000 | 0.020 | 0.000 |
| 8 |  |  | 0 | 0 | 0 | 0 | 0.000 | 0.000 | 0.000 | 0.000 |
| 8 |  |  | 0 | 0 | 0 | 0 | 0.000 | 0.000 | 0.000 | 0.000 |
| 8 |  |  | 0 | 0 | 0 | 0 | 0.000 | 0.000 | 0.000 | 0.000 |
| 8 |  |  | 0 | 0 | 0 | 0 | 0.000 | 0.000 | 0.000 | 0.000 |
| 8 |  |  | 0 | 0 | 0 | 0 | 0.000 | 0.000 | 0.000 | 0.000 |

| Group | Immunization Schedule (Study Days) | AV7909 Dilution | ED50 | | | | NF50 | | | |
| --- | --- | --- | --- | --- | --- | --- | --- | --- | --- | --- |
|  |  |  | Time Point | | | | Time Point | | | |
|  |  |  | Day -7 | Day 27 | Day 42 | Day 69 | Day-7 | Day 27 | Day 42 | Day 69 |
| 9 | 0,28 | Normal Saline | 0 | 0 | 0 | 6 | 0.000 | 0.000 | 0.000 | 0.007 |
| 9 |  |  | 0 | 0 | 0 | 6 | 0.000 | 0.000 | 0.000 | 0.007 |
| 9 |  |  | 0 | 0 | 0 | 4 | 0.000 | 0.000 | 0.000 | 0.005 |
| 9 |  |  | 0 | 0 | 0 | 0 | 0.000 | 0.000 | 0.000 | 0.000 |
| 9 |  |  | 0 | 0 | 0 | 0 | 0.000 | 0.000 | 0.000 | 0.000 |
| 9 |  |  | 0 | 0 | 0 | 0 | 0.000 | 0.000 | 0.000 | 0.000 |
| 9 |  |  | 0 | 0 | 0 | 0 | 0.000 | 0.000 | 0.000 | 0.000 |
| 9 |  |  | 0 | 0 | 0 | 0 | 0.000 | 0.000 | 0.000 | 0.000 |
| 9 |  |  | 0 | 0 | 0 | 0 | 0.000 | 0.000 | 0.000 | 0.000 |
| 9 |  |  | 0 | 0 | 0 | 5 | 0.000 | 0.000 | 0.000 | 0.006 |
| 9 |  |  | 0 | 0 | 0 | 0 | 0.000 | 0.000 | 0.000 | 0.000 |
| 9 |  |  | 0 | 0 | 0 | 4 | 0.000 | 0.000 | 0.000 | 0.005 |
| 9 |  |  | 0 | 0 | 0 | 0 | 0.000 | 0.000 | 0.000 | 0.000 |
| 9 |  |  | 0 | 0 | 0 | 0 | 0.000 | 0.000 | 0.000 | 0.000 |
| 9 |  |  | 0 | 0 | 0 | 0 | 0.000 | 0.000 | 0.000 | 0.000 |
| 9 |  |  | 0 | 0 | 0 | 0 | 0.000 | 0.000 | 0.000 | 0.000 |
| 9 |  |  | 0 | 0 | 0 | 0 | 0.000 | 0.000 | 0.000 | 0.000 |
| 9 |  |  | 0 | 0 | 0 | 0 | 0.000 | 0.000 | 0.000 | 0.000 |
| 9 |  |  | 2 | 0 | 0 | 0 | 0.006 | 0.000 | 0.000 | 0.000 |
| 9 |  |  | 0 | 0 | 0 | 0 | 0.000 | 0.000 | 0.000 | 0.000 |
| 9 |  |  | 0 | 0 | 0 | 0 | 0.000 | 0.000 | 0.000 | 0.000 |
| 9 |  |  | 0 | 0 | 0 | 0 | 0.000 | 0.000 | 0.000 | 0.000 |
| 9 |  |  | 0 | 0 | 0 | 0 | 0.000 | 0.000 | 0.000 | 0.000 |
| 9 |  |  | 0 | 0 | 0 | 0 | 0.000 | 0.000 | 0.000 | 0.000 |

**Supplemental File 2. Neutralizing Titer (TNA) Results of Each Individual Animal at Each Analyzed Time Point**

NS = No Sample Available
